# Health Care Payments in Vietnam: Patients’ Quagmire of Caring for Health versus Economic Destitution

**DOI:** 10.3390/ijerph14101118

**Published:** 2017-09-25

**Authors:** Andre Pekerti, Quan-Hoang Vuong, Tung Manh Ho, Thu-Trang Vuong

**Affiliations:** 1 Business School, The University of Queensland, Brisbane, QLD 4072, Australia; a.pekerti@uq.edu.au; 2 Centre for Interdisciplinary Social Research, Western University Hanoi (ĐH Thành Tây), Hanoi 100000, Vietnam; tung.ho@wu.edu.vn; 3 Centre Emile Bernheim, Université Libre de Bruxelles, 1050 Brussels, Belgium; 4 Institute of Philosophy, Vietnam Academy of Social Sciences, Hanoi 100000, Vietnam; 5 Sciences Po Paris—Campus de Dijon, 21000 Dijon, France; thutrang.vuong@sciencespo.fr

**Keywords:** health care, user fees, place of residence, insurance cover, Vietnam

## Abstract

In the last three decades many developing and middle-income nations’ health care systems have been financed via out-of-pocket payments by individuals. User fees charges, however, may not be the best approach or thenmost equitable approach to finance and/or reform health services in developing nations. This study investigates the status of Vietnam’s current health system as a result of implementing user fees policies. A recent mandate by the government to increase the universal cover to 100% attempts to tackle inadequate insurance cover, one of the four major factors contributing to the high and increasing probability of destitution for Vietnamese patients (the other three being: non-residency, long stay in hospital, and high cost of treatment). Empirical results however suggest that this may be catastrophic for low-income earners: if insurance cover reimbursement decreases below 50% of actual health expenditures, the probability of Vietnamese falling into destitution will rise further. Our findings provide policy implications and directions to improve Vietnam’s health care system, in particular by ensuring the utilization of health services and financial protection for the people.

## 1. Introduction

Recent trends in many countries indicate that user fees have been introduced in health care to help governments manage the cost of their health care systems. Such policies were in fact recommended by The World Bank in its 1986 document, “Financing Health Services in Developing Countries: An Agenda for Reform” [1]. The paper proposed that a user fees system would enable lower-income countries to focus on providing low-income earners with essential services [2,3,4]. It also recommended that the implementation of these policies should be supported by four main systems, namely social insurance, employer-based schemes, prepayment schemes, and private insurance [1].

Notwithstanding the idealistic intentions of The World Bank’s 1986 policy, the introduction of user fees systems has resulted in high medical costs for individuals in many nations. Research and data in the last three decades suggest that user fees charges may not be the best approach or most equitable approach to finance and/or reform health services in developing nations. In short, low-income earners, even those in upper-middle-income nations, will end up receiving proportionally less health care [5,6,7,8,9,10,11,12,13]. Furthermore, it has caused an unforeseen negative side effect: a poorer collective health for all due to the reducing access to health care [4,6,14,15]. Whitehead et al. even noted that as far back as July 2000 the US House of Representatives lobbied The World Bank to stop requesting that developing nations implement a user fees system [4].

On the other hand, out-of-pocket (OOP) is the most important means of financing health care in many nations, regardless of whether the country is developed or developing [11,12,13,16,17,18,19,20,21]. Numerous health care systems in upper-middle-income nations such as China [7,15,16,17] have been financed via OOP payments by individuals [18]. Van Doorslaer et al. (2006) in an 11-nation study showed that the pervasiveness of poverty in these nations was 14% above the conventional estimates when OOP payment for health care is taken into account [20]. This also applied to developing nations such as Vietnam: despite the universal insurance coverage and the recent policy of 100% reimbursement, rigid residency-based regulations and inadequate provincial infrastructures [21] led to a large number of non-resident patients in large cities becoming ineligible for significant insurance coverage [22]. In light of this, OOP payments only further exacerbate medical costs all while being regarded as a sensitive issue in transition economies like Vietnam and China, where health care infrastructures are inadequate and underinvested [21]. In Vietnam, paying informal thank-you money was widespread as subtly bribing health personnel in hopes of a better treatment became an unwritten rule amongst patients [22]. As our study is based on Vietnam, we define OOP payments as unofficial spending to cover these subtle bribes.

The issue of high health care in emerging nations such as Vietnam must be highlighted, as it often resulted in inadequate access to health care services. Expenditures have always been a key factor in Vietnamese people’s decisions regarding health care. For example, previous findings show that while periodic health examinations are usually overlooked and shunned as unnecessary extra costs [23], cash subsidy can significantly encourage people to take periodic health check-ups [24]. This economical mindset extends from the general population to special communities such as co-located patients clusters which were born from and held together by the needs to fight financial hardships of chronic, poor patients [25]. At the same time, it has been reported that patients who considered patients who were more pessimistic about the current state of public health were also more satisfied with the quality of the medical service they chose, suggesting that society made different health care choices based on diverging perceptions of collective health [26].

One of the issues driving this study is the fact that lower-income households are incredibly vulnerable in emerging economies, especially when their still-young populations were rapidly aging. Studies have shown that in poor rural households, the elderlies were obliged to work after retirement age due to inadequate incomes or lack of psychological health support [27,28,29]. Low-income households also face an ever-present risk of destitution if members of the household become sick [4,8,10,11,17]. In Thailand, for low-income households, spending approximately 70% of a household’s per-capita monthly income or spending 10% to 25% of household income per year would lead to household destitution [12,13,14]. Lower-income households are more likely to spend a larger proportion of their household income on health care compared to higher-income households [10,12]. Research further indicate that for some it is not a single disastrous event that leads to destitution but rather a series of every-day-illnesses and/or on-going care and/or payments that lead to destitution [8,9,12]. As such, in this study, a patient is considered to be in destitution when their financial distress due to treatment expenditure was drastic with debts that surpassed their income and were thus unpayable.

Since there has been little research about the probability of patients falling into destitution and related factors that contribute to the level of risk patients have to take on when deciding to use health care services [21], the purpose of this study is to investigate how user fees charges policies have impacted a lower-income nation, namely, Vietnam [30]. In particular, the study aims to provide more in-depth findings on the subject of destitution among patients, unofficial payments in health care, as well as factors affecting medical treatment outcomes. The factors taken into consideration include: accommodation, healthcare costs, income, hospital stay, heath insurance and severity of illness. Insights drawn would assist Vietnamese policy makers towards appropriate directions for improvements.

## 2. Materials and Methods 

### 2.1. Research Questions

To achieve the aims of understanding the current Vietnamese health sector and patients’ risks, we pose a number of research questions (RQ) to cull out nuanced knowledge to improve current practices and policies in Vietnam:

- RQ1. Evaluate the impact of residency status, length of hospital stay, health insurance, illness status and medical costs on the likelihood of a patient falling into destitution after treatment.
- RQ2: Are the practice of providing unofficial payment or tips (a form of OOP payments) to health practitioners and the amount of such payment determined by the severity of illness and/or income of patients?
- RQ3: Does higher cost result in more successful treatment outcomes? Compared to wealthier patients, do poor patients face disadvantages during treatment?

### 2.2. Dataset and Variables

The survey was conducted by a Hanoi-based research firm including hospital personnel from inpatients of hospitals in northern Vietnam. The northern area of Vietnam was chosen due to its great number of major hospitals, which could represent for the medical sector generally. Moreover, because of their location being near the capital city of Hanoi, hospitals in the northern tend to obey medical principles and laws more strictly. Some of hospitals were chosen such as Viet Duc Hospital, Bach Mai Hospital, Vietnam-Japan Hospital, Hai Duong Polyclinic Hospital, Thai Binh Polyclinic Hospital, and Ministry of Transport Polyclinic.

Interviews were conducted on all patients present at the time of the survey without discriminations, rules or criteria. Interviewers approached patients individually and acquired information for the survey, including questions about sensitive data that a more general/social survey cannot obtain. Questions comprised of family status, patient’s income level, patient’s extra expenses to doctors and hospital staff, including borrowing money to finance treatment; the procedures received ethical clearance. The research team obtained 1042 qualified observations. Data was then entered into a Microsoft v. 2010 Excel spreadsheet (Microsoft, Redmond, WA, USA), saved as a CSV file, and analyzed using the statistical software R (v 3.3.1; Bell Labs, Murray Hill, NJ, USA).

We acknowledge that the dataset present certain limitations, primarily geographical due to the fact that the data was gathered uniquely in hospitals and clinics in the north of Vietnam, and may not reflect attributes that are unique to other regions. A nationwide study of the same nature may show shifting tendencies due to regional differences. As of present, such a study is out of our capacity due to limited resources. The variables used in our study will be listed below, along with a short description where necessary:

- “Burden” represents the effect of health care expenditures on the financial situation of the patient as assessed by the patient himself, with three categories: “A” (Minimally affected), “B” (Adversely affected), and “C” (Destitute);
- “End” measures the success of a treatment as perceived by the patient, after treatment has ended. The variable admits four categories: “A” (Complete recovery), “B” (Self-care at home), “C” (Stopped), and “D” (Early quit);
- “Res” represents the residency status of the patient, admitting two values: “Yes” and “No”;
- “Insured” represents the insurance status of the patient, with two values: “Yes” and “No”;
- “Pins” is a continuous variable measuring insurance coverage, more precisely the percentage of insurance reimbursement on cost of treatment. This variable was not used directly in our analysis, but its values were used to constitute the next variable;
- “InsL” indicates the level of insurance coverage and consists of four categories: “Nil” (no imbursement, Pins = 0), “Low” (Pins ≤ 0.25), “Med” (Pins > 0.25 and Pins ≤ 0.45), and “Hi” (Pins > 0.45);
- “Illness” represents the seriousness of the patient’s illness in three categories: “Emerg” (Emergency), “Bad” (Rather severe) and “Light” (Medium or light);
- “AvgCost” measures the average daily treatment costs at three levels: “Hi” (>VND5.4 mn), “Med” (from > VND1.5 mn to VND5.4 mn), and “Lo” (VND1.5mn and lower);
- “Stay” indicates the duration of hospitalization, with two categories: “Long” (≥10 days) and “Short” (<10 days);
- “EnvL” is categorized into three levels according to the proportion of extra thank-you-money in medical costs that inpatients have to pay: “High” (>15%), “Med” (from >7% to 15%) and “Neg” (negligible, 7% and lower);
- “Income” is a continuous variable representing the average income per year (million VND) of the patient. This variable was not used directly in our analysis, but its values were used to constitute the next variable;
- “IncRank” consists of two levels of patient income: “Lo” (low, less than or equal to VND48 mn/year), and “HM” (medium-high, more than VND48 mn/year).

### 2.3. Methodology

Because most variables in this study are discrete, multi-category logit models (polytomous logistic regression analysis) were used to investigate the RQ1–3; the resulting models show behaviors of multinomial response variable (Y) following multinomial (and binomial) predictor variables. Subsequent analyses employed baseline-category logits (BCL). This type of modelling enables us to detect relationships between discrete variables, and in our survey, likely polytomous response variables and discrete (multinomial or binomial) explanatory variables. In addition, it allows us to compute useful probabilities upon specific events of hypothetical influence.

Now, let *π_j_*(**x**) = *P*(*Y* = *j*|**x**) represent a fixed setting for predictor variables, with ∑*_j_π_j_*(**x**) = 1. Count data are grouped into *J* categories of *Y* as multinomial with corresponding sets of probabilities {*π*_1_(**x**),…, *π_j_*(**x**)}.

The baseline category logit models align each response (dependent) variable with a baseline category, taking the form:

Ln(*π_j_*(**x**))/(*π_j_*(**x**)) = ***α****_j_* + ***β****_j_*’**x**, *j* = 1,…,*J*−1 (1)

A rich account of technical details for practical modelling of polytomous logistic models is provided in Agresti [31]. Actual estimations performed in this study–whose results are reported in the next sections—employ analysis in R, following a set of instructions provided by Penn State [32].

Since the main purpose of the estimation is to compute response probabilities from multinomial logits, i.e., {*π_j_*(*x*)}, the following computation will apply:

*π_j_*(**x**) = exp(***α****_j_* + ***β****_j_*’**x**)/(1 + ∑*^J^*^−1^_(*h* = 1)_ exp(***α****_h_* + ***β****_h_*’**x**)) (2)

with ∑*_j_*π*_j_*(**x**) = 1; *α_j_*= 0 and *β_j_*= 0. The computed probabilities can be used to model the risk of a patient falling into a category of financial distress (indebtedness or destitution) conditional upon some other events such as being in the lower socio-economic status group (SES) and/or being non-resident as to where the hospital is located, and/or being insured, and so on.

## 3. Results

In this section, we present eight estimations with significant coefficients which are reported in separate analyses. In each analysis coefficients are tabulated, followed by equation forms for stylized facts. Estimated probabilities are computed for the event conditional upon selected events specified by the related factors (predictors).

We first start with a section detailing some descriptive statistics from the dataset. The analyses from Section 3.1 to Section 3.5 are executed in order to answer question RQ1, which aims to evaluate probabilities of patients falling into destitution after experiencing treatment. The response variable of “Burden” (financial burden might affect patients and their family) is used for all models, including 3 categories which are labelled as A (Minimally affected—unaffected), B (Moderately affected—affected but manageable) and C (Destitute—not able to pay off medical loan).

### 3.1. Descriptive Statistics

**Figure 1:**
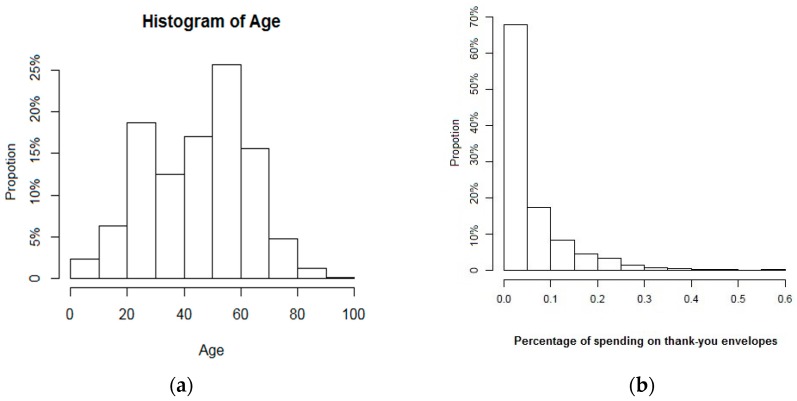
Distribution of patients towards (**a**) age and (**b**) the proportion of thank-you money in total treatment fee.

As it can be seen in Figure 1a, the majority of participants are from 20 to 70 years old (87%) and the oldest is about 90. Figure 1b show that approximately 90% of patients pay less than 15% of the total expenses for thank-you envelopes, implying that thank-you envelopes greater than 15% of total expense as a threshold is considered to be an unaffordable amount of OOP payment.

Table 1 shows approximately 76% of patients have incomes lower than approximately U.S. $2315 (about VND48 mn) per year, and 90% have an annual income of below U.S. $4630. In other words, 76% of patients in our sample represent the average lower- to middle-income earners of Vietnam (The World Bank, 2015; since Vietnam has a per capita gross national income of U.S. $1890 in 2014). Out of 1042 participants, over 80% have been hospitalized for 10 days or less. Specifically, nearly 3/5 of them stay in from 5 to 10 days, additionally, most of them are poor. Table 1 also shows that most of patients have to pay treatment cost from >VND1.5 mn to VND5.4 mn and having a total treatment fee of VND50 mn or less (nearly 85%). The proportion of patients paying more than VND100 mn only accounts for about 3.5%.

It should also be remarked that while the majority of patients had low incomes, the length of hospitalization and the average cost were low to medium: over 46% of all patients were of low income (under VND48 million per year) and were hospitalized between 5 and 10 days. Lower-income patients also had significantly fewer short stays than other income groups; in fact, while only over 18% low-income patients spend less than 5 days hospitalized (13.29 out of 76.10 percentage points), at least nearly one-third of middle- and high-income patients (7.01 out of 23.13 and 0.29 out of 0.77 percentage points, respectively) had such short hospital stays.

On another note, there were a higher percentage of long stays, high average costs and high treatment fees among high income patients. Specifically, nearly 25% high-income patients spent over 10 days in the hospital (0.19 out of 0.77 percentage points) as opposed to only about 19% of the entire sample having such long stays. Similarly, almost 13% high-income patients afforded a total treatment cost of over VND100 million (0.1 out of 0.77 percentage points) while in general only over 3.5% of patients paid the same amount, as mentioned above.

**Figure 2:**
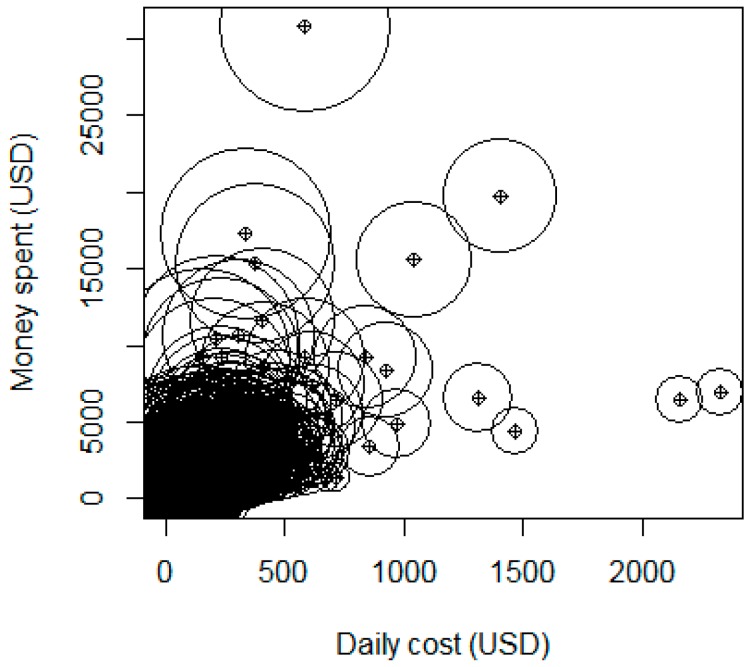
Daily total expenses in USD and daily cost in hospital.

Figure 2 represents data points, each with three numerical values of average daily cost (horizontal axis in U.S. dollar per day) in relation to the total health care expenses for the treatment (vertical axis; in U.S. dollar) and number of days in the hospital (taking the natural logarithm to reduce the difference in effect size for better visualization). The differences among patients are quite substantial. If patients have to pay a significant amount daily, they will soon quit the treatment. A large proportion of this sample spent long time in hospital with average cost less than U.S. $500 per day (bigger circle, concentrate near the origin of the axis).

**Figure 3:**
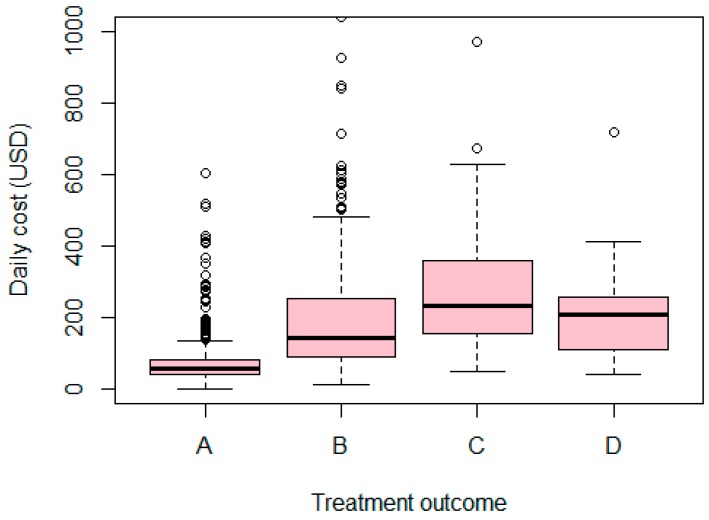
Treatment outcomes in relation to average daily cost. Treatment outcomes. A—Full recovery; B—Partial recovery; C—Stopped in middle; D—Unsuccessful treatment, including mortality.

Figure 3 illustrates average daily cost per patient, divided into groups of patients with different treatment outcomes. Income is on the vertical axis, measured in VND. Figure 3 indicates that patients in group C who have to quit their treatment mid-way and patients in group D who have an unsuccessful treatment have a higher average daily cost than the others. The successfully treated group (group A) has the lowest average daily cost. Notably, the most expensive cost per day is found for patients in group B—patients who had a partial recovery.

### 3.2. Joint Effects of Residency and Insurance Status

The logistics regressions enable us to analyse the joint effects of a patients’ residency status (“Res”) and insurance status (“Insured”) to estimate the likelihood that patient will be adversely affected by health care expenditure (“Burden”). In this estimation, the baseline is the minimally affected category (A). The references for both independent variables “Res” and “Insured” are “Yes”. Results are provided in Table 2 with all coefficients being statistically significant at a conventional level (*p* < 0.001).

The regressions of Equations (3) and (4) are established based on Table 2 to display the correlations between the variables:

ln(*π̂*_C_/*π^*_A_) = −2.583 + 3.812 × no.Res + 1.663 × no.Insured (3)

ln(*π̂*_B_/*π^*_A_) = −1.290 + 1.782 × no.Res + 1.601 × no.Insured (4)

As it can be observed in Equation (3), the coefficient of “Res” is larger than which of “Insured”, therefore the factor of residency status will have a stronger effect on the probability of destitution. The two above equations can be used to calculate the likelihood of being adversely affected by the health care cost, for a patient who has negligible to no insurance and is a non-resident of a region (or from a faraway province). In fact, there is a nearly 30% chance that such a patient would be burdened by their health care expenditure. The formula was as follows:

*π̂*_B_ = e^−^^1.290 + 1.783 + 1.601^/(1 + e^−^^2.583 + 3.812 + 1.663^ + e^−^^1.290 + 1.783 + 1.601^) = 0.299 (5)

The probability of this same person becoming destitute is over 66%, computed in the following formula:

*π̂*_C_ = e^−^^2.583 + 3.812 + 1.663^/(1 + e^−^^2.583 + 3.812 + 1.663^ + e^−^^1.290 + 1.783 + 1.601^) = 0.664 (6)

And the chance of this person being minimally affected amounted to 3.7%, according to our calculations:

*π̂*_A_ = 1 − 0.299 − 0.664 = 0.037 (7)

The same methods were applied to the other conditional probabilities, shown in Table 3.

**Figure 4:**
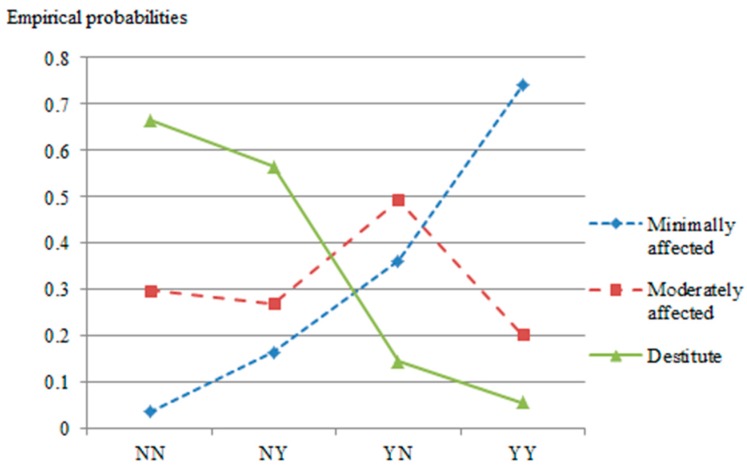
Contrasting financial welfare of patients as a function of status of residency and insurance status. NN—non-resident & uninsured; NY—non-resident & insured; YN—Resident & uninsured; YY—Resident & insured.

The joint effects of a patients’ residency status and their insurance status indicate that those most at risk are patients who are non-residents of a region (or hereforth, from another province) and uninsured, at 66.4% chance of becoming destitute as a result of health expenditure. Most striking, only less than 4% of such patients—uninsured and from another province—would escape financial distress. Figure 4 shows trends of changing probabilities among different group of patients characterized by status of residency and by eligibility to health insurance coverage. 

The diamond dotted line indicates how changes in the effects of residency and insurance status improve patients’ financial welfare depending on insurance and residency status. It can be seen that the probability line of “Destitute” goes downward whereas “Minimally affected” goes upward from NN (non-resident & uninsured) to YY (resident & insured). Therefore, being both non-resident and uninsured amplified the risk of falling into post-treatment destitution.

### 3.3. Joint Effects of Residency and Levels of Insurance Reimbursement

Our next estimation models the probability of falling into a specific category of post-treatment financial position, conditional upon insurance reimbursement levels (“InsL”) and the residency status of the patient (“Res”) and is based on the dataset. Results are provided in Table 4.

From estimated coefficients in Table 4, Equations (8) and (9) are built to perform the relationships between residency status, medical payment level from insurance reimbursement and financial burden:

ln(*π̂*_C_/*π̂*_A_) = −2.724 + 3.709 × no.Res + 1.384 × low.InsL + 0.129 × med.InsL + 1.967 × nil.InsL (8)

ln(*π̂*_C_/*π̂*_A_) = −1.359 + 1.721 × no.Res + 1.047 × low.InsL + 0.080 × med.InsL + 1.780 × nil.InsL (9)

The results show a trend where having no insurance and being a non-resident increases the chances (log-odds) of becoming indebted, namely moderately affected and/or destitute by health expenditure. From Equations (8) and (9) we can calculate probability of Destitution (*π^*_C_), and being Moderately affected (*π* ^_B_) as a function of residency status and the level of payment from insurance reimbursement. 

Further modelling (Table 4) enable us to measure the risks of being financially affected due to health expenditure as well as in relation to residency status and insurance reimbursement levels. For example, using data from the category of Adversely affected/Destitute indicates that non-residency factor has a much larger (negative) effect on the probability of a patient becoming indebted compared to being uninsured; the significant coefficient being 3.709, compared to 1.967, respectively (Table 5). The probability of a patient without insurance coming from another province and becoming indebted is as high at 67%.

**Figure 5:**
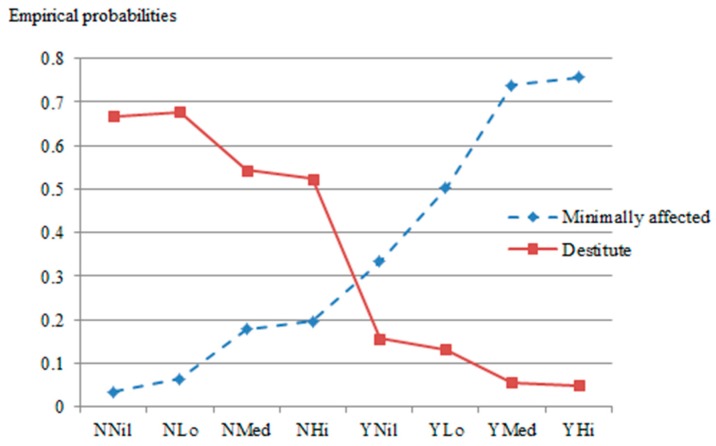
Contrasting financial risk as function of status of residency (N/Y) and actual insurance cover (nil/low/med/high). N—non-resident; Y—resident; Nil—uninsured; Lo—low insurance cover (low insurance reimbursement), Med—medium insurance cover (medium insurance reimbursement); Hi—high insurance cover (high insurance reimbursement).

Regardless of whether or not a patient has insurance coverage, the risk of one being destitute due to health care expenditure if one receives treatment in a different province is very high, starting from 52% to 67%. In contrast, for a person with no insurance yet is a resident of the province where they receive treatment, the highest chance of facing destitution falls to 16%. The weight of being a non-resident in a region on one’s financial burden is shown in Figure 5. It can be seen that the probability line of “Destitute” has a downward trend when moving from “No” to “Yes” of “Res” and from “Hi” to “Lo” of “InsL”, while the opposite trend occurs for “Minimally affected” line. In other words: The general trend is that having residency status and higher insurance reimbursement contribute to reducing financial distress; more than that, lack of residency status has a much more perverse effect on financial welfare than lack of insurance.

### 3.4. Joint Effects of Health Care Cost and Severity of Illness

The upcoming analyses will focus on clarifying the change of probabilities of a patient being under financial burden conditional on the status of the patient’s illness (“Illness”) and the average daily cost (“AvgCost”).

The estimated coefficients of the logistic regression model with the response “Burden“ and the predictors “Illness“ and “AvgCost“ are provided in Table 6. All the coefficients are statistically significant with *p* < 0.01. From that the two below regression equations are established:

ln(*π̂*_C_/*π̂*_A_) = −3.733 + 1.002 × bad.Illness + 1.756 × emerg.Illness + 4.790 × hi.AvgCost + 3.470 × med.AvgCost (10)

ln(*π̂*_B_/*π̂*_A_) = −1.777 + .658 × bad.Illness + 0.828 × emerg.Illness + 2.275 × hi.AvgCost + 1.664 × med.AvgCost (11)

The coefficient of “AvgCost” is larger than that of “Illness”, which indicates a strong influence of “AvgCost” on patients. *π̂*_A_, *π̂_B_*, *π̂*_C_ calculated from Equations (10) and (11) show that the probability of a patient who suffers a severe illness and has to pay at a high level of medical fee being indigent is very high, amounting to nearly 78%. Likewise, the probability of a person who has a light disease and pays at a low level being minimally affected is about 84%. Table 7 shows us actual empirical computations of related probabilities.

**Figure 6:**
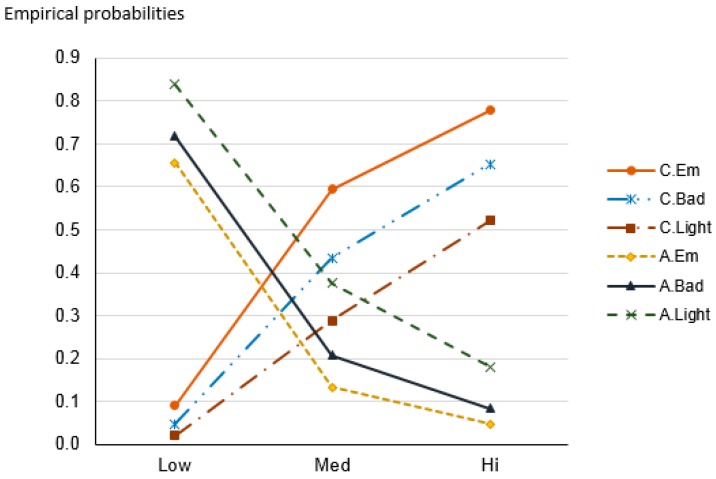
Contrasting risks as a function of illness and average cost of treatment. C—Destitute, A—Minimally affected; Em—Emergency; Bad—Bad-illness; Light—Light-illness.

Figure 6 presents trends of these changing probabilities, drawn upon selected values of Table 7.

The contrast is continuously repeated in Figure 6. All the lines representing for the probabilites of a patient falling into destitution (“C.Em”, “C.Bad”, và “C.Light”) go up when moving from “Low” to “Hi”. Conversely, the lines represent for patient being minimally affected (“A.Em”, “A.Bad”, và “A.Light”) all go down. Therefore, higher medical cost will increase the probability of a patient being destitute. Regarding the relative positions of these lines, “C.Em” being significantly above “C.Bad” and “C.Light” implies that patients with more severe illnesses are more likely to be destitute after receiving treatment.

### 3.5. Effect of Long vs. Short Stay in Hospital to Patients’ Financial Outcome

We now estimate the effect of the duration of hospitalization (“Stay”) on financial outcome (“End”). Our estimates of patients from another province who has to stay more than 10 days in hospital without insurance can only minimally affect financially show a bleak outlook. The results of the regression model with the dependent variable “Burden” and the independent “Stay”, “Res” and “Insured” are presented in Table 8, with all coefficients being statistically significant at any conventional level.

From the results of regression shown in Table 8, we create a model that incorporate effect of length of stay in hospital are provided below:

ln(*π̂*_C_/*π̂*_A_) = −2.885 + 1.230 × long.Stay + 3.651 × no.Res + 1.728 × no.Insured (12)

ln(*π̂*_B_/*π̂*_A_) = −1.428 + 0.757 × long.Stay + 1.682 × no.Res + 1.600 × no.Insured (13)

The duration of stay has, in fact, the least significant impact on the financial outcome of a patient, with the coefficient of “Stay” being the smallest. On the other hand, residency status has a strong effect on the probability of a patient bearing financial burden after experiencing treatment, *β*_2_ = 3.651 (*p* < 0.001) being the largest coefficient.

Complete distributions of empirical probabilities are given in Table 9, showing that insurance generally improves financial positions for both resident and non-resident patients. It is worth noting that when they are hospitalized for more than 10 days, an uninsured non-resident patient has an extremely low probability (1.79%) of becoming minimally affected financially after treatment, while resident patients tend to be in better financial states. 

**Figure 7:**
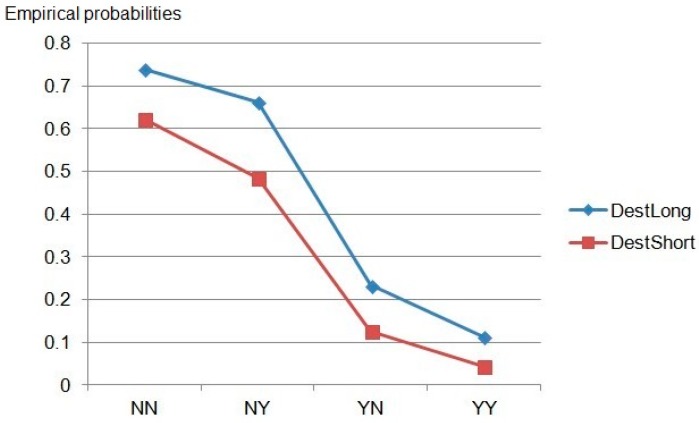
Changing probabilities of destitution for patients as a function of short versus long hospitalization. NN—non-resident & uninsured; NY—non-resident & insured; YN—Resident & uninsured; YY—Resident & insured.

Figure 7 illustrates a trend of changing probabilities of destitution for patients as a function of short versus long hospitalization. (*Nonresident-No insurance* to *Resident-Insured*). In short, Figure 7 shows that staying longer in hospital significantly increases the risk of facing destitution.

### 3.6. Influence of Factors on a Patient’s Probability of Falling into Destitution in Comparison

The results from Section 3.2 to Section 3.5 indicate that residency status, health insurance status, level of insurance reimbursement, average daily cost, illness status and length of hospital stay all had an impact on the risk of falling into post-treatment destitution among patients. In order to determine the most powerful factor, a regression model is employed with all the above factors of “Res”, “Insured”, “InsL”, “AvgCost”, “Illness” and “Stay” being predictors. The estimation result is provided in Table 10.

From Table 10, it can be observed that 3/4 of the coefficients are statistically significant, excepting those of “Insured”. The following equations are built using these coefficients above:

ln(*π̂*_C_/*π̂*_A_) = 3.122 − 1.227 × short.Stay − 2.644 × yes.Res + 0.123 × yes.Insured + 1.536 × lo.InsL + 0.254 × med.InsL + 2.224 × nil.InsL − 3.365 × lo.AvgCost − 1.584 × med.AvgCost + 0.254 × emerg.Illness − 0.992 × light.Illness (14)

ln(*π̂*_B_/*π̂*_A_) = 2.065 − 0.622 × short.Stay − 1.113 × yes.Res − 0.283 × yes.Insured + 0.982 × lo.InsL + 0.138 × med.InsL + 1.524 × nil.InsL − 1.466 × lo.AvgCost − 0.732 × med.AvgCost − 0.230 × emerg.Illness − 1.003 × light.Illness (15)

In Equation (14), β_7_ at “Lo” of “AvgCost” is the coefficient that has the largest absolute value. This means that, interestingly, low average daily cost is the strongest boost on the probability a patient being in severe financial distress after receiving treatment. Residency status is the second most powerful factor, with *β*_2_ = −2.644 (*p* < 0.001).

### 3.7. On the Sensitive Issue of Extra Thank-You Money (Envelope—OOP Payment)

In order to resolve the issue in RQ2, we aimed to find the probability of a patient paying high or medium extra thank-you money (“EnvL”) conditional upon income ranks (“IncRank”) and/or severity of illness (“Illness”). For Illness, the reference category is “Light”; for Income Rank, the reference is HM. 

The majority of estimated coefficients in Table 11 are statistically significant with *p* value < 0.01. From that, the relationships between the variables can be presented in Equations (16) and (17) as follows:

log(π̂_Hi_/π̂_Neg_) = −0.254 − 2.106 × BadIllness − 1.102 × EmergIllness − 0.896 × LowIncRank (16)

log(π̂_Med_/π̂_Neg_) = −0.555 − 0.582 × BadIllness − 0.316 × EmergIllness − 0.903 × LowIncRank (17)

It can be observed that all coefficients are negative, which shows that the category *Low-Income Rank* and both *Bad* and *Emergency of Illness* jointly reduce the probability of patients paying *thank-you money* from medium to high level. In Equation (16), the category of “Bad” of “Illness” has the largest absolute value (*β*_1_ = −2.106, *p* < 0.001). This means that the status of severe illness have the strongest impact on the probability of high thank-you money.

Table 12 is established by computing the conditional probabilities based on Equations (16) and (17). From that, there are approximately 8.25% lower-income patients who are willing to make an expensive extra *thank-you* OOP payment (π̂_Hi_), when they have an emergency-illness, while 13.3% of patients are willing to pay moderate amounts of extra *thank-you* OOP payment (π̂_Med_). However, 78.45% of low-income patients facing an emergency-illness can only afford a negligible amount of *extra thank-you* OOP payment.

Table 12 summarizes the distribution of probabilities for two groups of patients (by income rank), following levels of OOP spending (Negligible/Medium/High) and severity of illness (Light/Bad/Emergency). Table 12 indicates that poor patients who are seriously ill tend to spend substantially less on *extra thank-you* OOP payment. In contrast, richer patients with less serious illness tend to spend more on *extra thank-you* OOP payment. The estimates show that richer patients are two times more willing to pay high *extra thank-you* OOP payment compared to a lower-income patient when they face an *emergency* and *bad-illness*.

### 3.8. Relationship between Treatment Outcome, Insurance Status and Average Cost

The analysis in Section 3.8 and Section 3.9 aims to answer RQ3. The dependent variable is “End”, representing treatment outcomes. The two independent variables include whether or not a patient is insured (“Insured”), and average daily medical cost (“AvgCost”). The results of this regression are shown in Table 13. The reference line of “Insured” is “Yes” (patients are partly/fully supported by insurance) and that of “AvgCost” is Low Cost (“Lo”).

The empirical relationships between the variables are obviously affirmed as all estimated variables are statistically significant with *p* value < 0.01. The estimation equations are as below:

ln(π̂_D_/π̂_A_) = −4.106 + 1.513 × No.Insured + 3.526 × Hi.AvgCost + 1.849 × Med.AvgCost (18)

ln(π̂_C_/π̂_A_) = −5.079 +1.488 × No.Insured + 4.564 × Hi.AvgCost + 2.462 × Med.AvgCost (19)

ln(π̂_B_/π̂_A_) = −1.716 + 0.772 × No.Insured + 3.119 × Hi.AvgCost + 1.757 × Med.AvgCost (20)

It can be seen that the estimation coefficient of “Hi” of “AvgCost” (*β*_2_) is the largest among the three above equations. Therefore, paying the average daily cost at a high level will have the most powerful influence on treatment outcomes. The conditional probability of a patient who could successfully finish the treatment in the *Complete recovery* category is 0.0667, a rather small figure. Table 14 provides complete distributions of these probabilities computed across various conditions as applicable.

**Figure 8:**
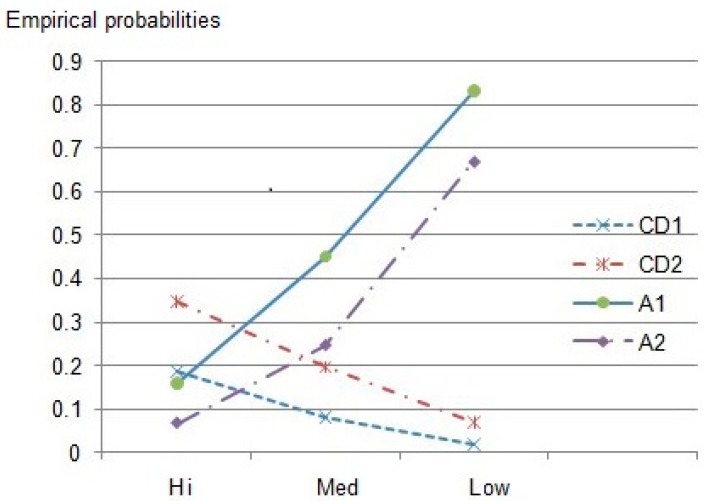
Comparative probabilities of treatment outcome by insurance status and average cost. Notes: CD1—Probabilities of Stopped and Early quit of insured patients; CD2—probabilities of Stopped and Early quit of uninsured patients; A1—probabilities of Complete recovery of insured patients; A2—probabilities of Complete recovery of uninsured patients

We can group both *Stopped* (Category C) and *Early quit* (Category D) into a single category of incomplete treatment and compare them with value of the *Complete recovery* category to see if there exists contrast to show the bigger picture. A more complete picture is depicted in Figure 8 which suggests that high cost makes it more likely that treatment is unsuccessful for patients who have no insurance. Thus it is logical that having some type of insurance increases the chances for all patients to recover in hospital.

### 3.9. Effect of Income on Treatment Outcome

The regression model is employed with “End” as the response variable and the “Income” as the predictor to analyse the effect of patients’ income per year (million VND) on their treatment outcome. Estimate results are provided in Table 15.

Based on that the three following estimation equations are built:

ln(π̂_A_/π̂_D_) = 1.5088 + 0.0175 × Income (21)

ln(π̂_B_/π̂_D_) = 1.7475 + 0.0031 × Income (22)

ln(π̂_C_/π̂_D_) = −0.9708 + 0.0183 × Income (23)

From the above equations, the probability that a patient earning VND100 mn per year on average obtains successful treatment is 70%. The calculation is as follows:

π̂_A_ = e^1.5088 + 0.0175^^×^^100^/(e^1.5088 + 0.0175^^×^^100^ + e^1.7475 + 0.0031 × 100^ + e^−0.9708 + 0.0183 × 100^) = 0.6993 (24)

The same procedures are applied to other conditional probabilities. The results are displayed in Table 16.

It is observed that when income increases, apparently the likelihood of successful treatment also rises (from nearly 40% to 88% as the income being maximum). Conversely, the probability of failed outcomes decreases when income grows, this figure, however, is rather small (less than 9%).

## 4. Discussion

Vietnam as a nation is categorized as a lower-middle income nation by The World Bank [30], and our data confirms this with 76% of our sample representing lower-middle income earners thus they do not live below the poverty line (Table 1). Unfortunately, as van Doorslaer et al. indicate, more than 14% of this 76% can be reclassified as lower-income earners when OOP payments for health care are taken into account [20]. Our research has uncovered a number of surprising statistics about the impact of health care costs on Vietnam’s population.

First, approximately 70% of patients face the risk of destitution. In other words, for every three uninsured and non-resident hospitalized patients, two are likely to face serious financial hardship or destitution. Our data further show that for every two patients hospitalized with a serious-illness that requires costly treatment, at least one is likely to face destitution.

A patients’ risk of being adversely affected by health care costs increases when they are either uninsured or not eligible for reasonable coverage. The effect is compounded if one is treated outside of one’s region of residency (or from a faraway province) with the estimated probability of falling into hardship at approximately 66% (Table 3).

Our data indicates that non-residency has a much larger (negative) effect on the probability of a patient becoming indebted compared to being uninsured. In particular, regardless of whether one has insurance or not, if one is a non-resident then the chances of becoming destitute is 52% to 67% (Table 5). Residency status is in fact the second-most influential factors regarding the risk of financial distress: it increases the probability of destitution. The only stronger predictor is low average daily cost, with the same negative influence. One explanation to this may be that most patients who pay low daily costs were low-income patients as well, which is strongly correlated to risk of destitution.

As the amended Law on Health Insurance comes into effect [33], the ambitious plan of aiming at 100% UC and all Vietnamese having health insurance is faced with large challenges. Although the implementation of UC plan has benefitted low-income households [18], the plan did not succeed in solving the issue of unequal access to health services [7]. For example, while the current statistics in this survey show that approximately 60% of Vietnamese hold UC, the majority of these insured patients are not adequately financed by insurance, (see Figure 2a,b); as noted above the insurance cover reimburses less than 50% of their actual expenditures. These figures suggest that if the current level of 60% insured increased to 100% as mandated by the Law on Health Insurance, then the actual coverage range and reimbursement each household would receive may decrease further below the current level of less than 50%; which is a daunting prospect. Our findings projects that if the insurance cover reimbursement decreases further than 50% of actual health expenditures to the point of negligible insurance level, then our estimates suggests that the probability of falling into destitution will rise.

Illness status is also proved to have a notable effect on post-treatment financial burden. The more severe the illness, the higher probability of patients facing financial risk, regardless of their high or low total treatment cost. For example, a patient whose average cost per day falls into medium range will have 29–60% of likelihood to fall into destitution when illness status changes from light to severe (Table 7).

Moreover, staying in hospital for a long time will cause them to face more difficulties. This is because having a multi-day time in hospital results in more treatment cost and extra fees. Thus, the likelihood being broke and indigent after treatment might rise to almost 74% (Table 9).

In addition, we believe that asymmetric information and lack of alternative financing should also be taken into account as a factor that can contribute to higher probabilities of destitution. Already, the poor only have a one-out-of-four chance of making the optimal choice concerning health service providers [34]. Our data show that a large portion of health care expenditures are caused by the problem of asymmetric information, exacerbated by poor patients’ borrowing, especially in the current situation in Vietnam where loan sharks are rampant and frequently used by lower-income patients/households. 

The analytical results also reveal negative influence when patients have to face with high treatment cost. This not only boosts the probability of destitution (Figure 6) but also reduce the chance of successful treatment outcome (<17%). Therefore, a good recovery depends on various factors such as the type of disease, the in-built feature of each person, etc. Bad outcomes usually have higher average treatment fees which may be due to their more severe illness status, leading to further diagnoses and longer hospital stay.

Furthermore, limitations in the treatment process of poor patients should also be called out. It implies that the poor lack access to medical services or receive low-quality ones, which leads to a lower chance of successful treatment. Previous studies have clearly documented that low-income households simply do not seek health care services because they cannot afford it [10,12], are reluctant to use them because these services are cost prohibitive [5,7,8,9,11] and/or delay treatment until they become incapacitated [8,35].

Contrary to popular beliefs, however, our analyses show that the majority of surveyed patients spent less than 5% of expenditures for this *extra thank-you money*. This suggests that the issue is more symbolic than substantial in relation to destitution. In fact, more than 85% of patients from lower-income households, who are seriously ill and anticipate costly treatment, pay negligible *extra thank-you money* during their hospital stay. At the same time, it should be noted that according to our results, wealthy patients with less serious illnesses are actually more likely to spend more *thank-you money* than poor and severely ill patients. The motivation for rich patients to spend more on *extra thank-you* OOP could be to avoid spending more time in hospital coupled with the expectation that they will receive better care from the nurses and doctors. In particular, if the illness is not serious, the *extra thank-you* OOP serves to ensure a faster diagnosis, such as faster processing of tests, better attitude and use of better equipment/medicines. Thus, another possible explanation would be that lower-income households who simply cannot pay more than a negligible amount of bribe [12] constitute the majority who paid little *thank-you money*.

It is noteworthy to mention that, despite our relatively large sample of 1042 patients, it has limitations, because it only sampled patients in the North Vietnam region. Therefore, future studies can improve on our current study and information relating to health services by sampling data from the middle and Southern parts of Vietnam. For example, studies show that health seeking behavior is moderated by culture and gender [5,35,36], where men generally delay treatment until they become incapacitated. Likewise, the elderly, and women also are highly vulnerable [19] and disadvantaged in terms of health care and in earnings [16], despite the fact that they are often the primary care givers.

In closing, we offer a number of policy implications based on our current and findings from previous studies where nuanced strategic policies have been found to be one of the solutions for health care systems [36]. In particular, we advocate that “strategic policy(ies) formation in all health care systems should be based on information relating to health promoting, seeking and utilization behaviour and the factors determining these behaviours” [36].

First, notwithstanding the benefits that low-income households retrieve from UC plans [18] and health insurance reforms [9], a user charge system without a targeted system of exempting payments from low-income earners will lead to destitution for many Vietnamese [7,18]. For example, even though the rates of absolute poverty have fallen in Vietnam, a series of health care cost related to “every-day-type” of illnesses can lead destitution for low-income households [37]. Research has shown that a targeted system of exempting payments from low-income earners will help low-income households [3,7,12,18]. We suggest that the Ministry of Health lobby for such plans to be implemented as a multiple step process; whereby a review of the system can be done once health care equity has reached a certain threshold for Vietnam.

Second, a variation to the above policy implication is targeting exemptions for particular illnesses that may incur high costs [14]. In this manner, we can target helping a proportion of the population who may be at most risk due to particular illnesses [38].

Third, moving away from out-of-pocket payments to prepayment mechanisms is the key to reducing catastrophic financial outcomes [12,13,19,20] and/or ensuring equity in health care services [21]. As such, better targeting policies might help government solve this problem, e.g., means testing.

Fourth, to support health insurance reforms and reduce the burden on state budget, employer-based cover systems can be implemented in conjunction with prepayment mechanisms [7,13]. Again the Ministry of Health should be able to lobby for such systems to be implemented, initially in large-scale organization, then to medium-scale organizations once the system has been established.

## 5. Conclusions

Our analyses provide the public and policy makers with a more nuanced understanding than is currently available. In short, we found the following factors contributing to the increase in the probabilities of destitution for the Vietnamese population:

1. Non-residency of patients is associated with two major issues: (a) travel costs, and (b) asymmetric information (i.e., lack of knowledge about medicine, methods of healthcare or their status of illness).
2. High costs of treatment, including equipment, drugs, care and room. This factor is also the most significant factor that leads to high probability of indebtedness faced by patients after treatment;
3. Inadequate insurance coverage: Although in theory, most patients with Vietnamese universal cover (UC) are entitled to 80% to 100% coverage, in practice that has not been achieved. The empirical data show that the majority of patients are reimbursed less than 50% of their actual expenditures, and finally,
4. The duration of stay in hospital significantly affects the financial position of patients after treatment.

Suggestions for policy-makers, derived from these conclusions, include: (1) a step-by-step revision of the current insurance law to exempt low-income households from obligatory payments; (2) targeting high-cost illnesses for payment exemptions; (3) prepayment as a solution to the issue of out-of-pocket payments; (4) employer-based systems to reduce burden on the government. Most of these policy implications should not be impossible to lobby for, especially if the Ministry of Health gets involved.

## Figures and Tables

**Table 1:** Distributions of patients towards their length of hospital stay, average daily cost and total treatment fee and average yearly income, unit: %.

Title	Average Yearly Income			
**Length of hospital stay**	≤VND48 mn/year	>VND48 mn/year and ≤VND180 mn/year	>VND180 mn/year	Total
>10 days	15.93	3.07	0.19	19.19
>5 days and ≤10 days	46.26	13.05	0.29	59.60
≤5 days	13.92	7.01	0.29	21.21
*Total*	*76.10*	*23.13*	*0.77*	**100**
**Average daily cost**	≤VND48 mn/year	>VND48 mn/year and ≤VND180 mn/year	>VND180 mn/year	Total
>VND5.4 mn	11.52	3.55	0.19	15.26
>VND1.5 mn and ≤VND5.4 mn	33.40	9.69	0.19	43.28
≤VND1.5 mn	31.19	9.88	0.38	41.46
*Total*	*76.10*	*23.13*	*0.77*	**100**
**Total treatment fee**	≤VND48 mn/year	>VND48 mn/year and ≤VND180 mn/year	>VND180 mn/year	Total
>VND100 mn	2.69	0.77	0.10	3.55
>VND50 mn and ≤VND100 mn	8.54	1.82	0.10	10.46
>VND20 mn and ≤VND50 mn	18.71	4.99	0.10	23.80
≤VND20 mn	46.16	15.55	0.48	62.19
*Total*	*76.10*	*23.13*	*0.77*	**100**

**Table 2:** Estimation for probability of distress on residency and insurance of patients.

	Intercept	“Res”	“Insured”
	*β*_0_	“No”	“No”
		*β*_1_	*β*_2_
Logit(C|A)	−2.583 ***	3.812 ***	1.663 ***
	[0.177]	[0.233]	[0.239]
	(−14.589)	(16.358)	(6.954)
Logit(B|A)	−1.290 ***	1.782 ***	1.601 ***
	[0.109]	[0.204]	[0.218]
	(−11.186)	(8.748)	(7.352)

Notes: Residual deviance: 18.73 on 2 degrees of freedom (d.f.); Log-likelihood: −29.86 on 2 d.f; baseline category for: “Res” = “Yes”, and “Insured” = “Yes”; (s.e.) and z-values in parentheses [] and (); *** *p* < 0.001.

**Table 3:** Summary of probabilities of destitution on residency and insurance of patients.

Resident	Insured	Financial Outcome		
		Minimally Affected	Moderately Affected	Destitute
**No**	No	0.0368	0.2988	0.6643
	Yes	0.1652	0.2702	0.5646
**Yes**	No	0.3619	0.4938	0.1442
	Yes	0.7403	0.2037	0.0559

**Table 4:** Modelling probabilities of financial distress upon residency and insurance of patients.

	Intercept	“Res”	“InsL”		
	*β*_0_	“No”	“Lo”	“Med”	“Nil”
		*β*_1_	*β*_2_	*β*_3_	*β*_4_
Logit(C|A)	−2.724 *** (−14.355)	3.709 *** (15.413)	1.384 *** (3.984)	0.129 (0.395)	1.967 *** (7.324)
Logit(B|A)	−1.359 *** (−11.629)	1.721 *** (8.148)	1.047 *** (3.296)	0.080 (0.265)	1.780 *** (7.439)

Baseline category for: “Res” = “Yes”, “InsL” = “Hi”; z-values in parentheses; *** *p* < 0.001. Residual deviance: 42.4228 on 6 degrees of freedom; Log-likelihood: −55.3995 on 6 degrees of freedom.

**Table 5:** Probabilities of financial distress upon residency and insurance of patients.

Resident	Insurance Level	Minimally Affected	Moderately Affected	Adversely Affected
**No**	Nil	0.0349	0.2972	0.6679
	Lo	0.0634	0.2594	0.6772
	Med	0.1786	0.2777	0.5437
	Hi	0.1956	0.2808	0.5235
**Yes**	Nil	0.3343	0.5090	0.1567
	Lo	0.5017	0.3670	0.1313
	Med	0.7392	0.2056	0.0551
	Hi	0.7562	0.1942	0.0496

**Table 6:** Modelling financial burden following cost levels and illness.

		“Illness”		“AvgCost”	
	Intercept	“Bad”	“Emerg”	“Hi”	“Med”
	*β*_0_	*β*_1_	*β*_2_	*β*_3_	*β*_4_
Logit(C|A)	−3.733 *** (−12.333)	1.002 *** (4.035)	1.756 *** (6.416)	4.790 *** (12.886)	3.470 *** (13.373)
Logit(B|A)	−1.777 *** (−8.941)	0.658 ** (3.154)	0.828 *** (3.368)	2.275 *** (6.634)	1.664 *** (9.232)

Residual deviance: 2172.53 on 8 d.f.; Log-likelihood: −49.92 on 8 d.f.; z-value in brackets. Baseline category for “Illness” = “Light”, “AvgCost” = “Lo”; *** *p* < 0.001, ** *p* < 0.01.

**Table 7:** Probabilities of financial burden following cost levels and illness.

Illness	Average Cost	Minimally Affected	Moderately Affected	Destitute
**Emergency**	High	0.0467	0.1757	0.7776
	Medium	0.1335	0.2728	0.5937
	Low	0.6556	0.2536	0.0908
**Bad**	High	0.0833	0.2644	0.6523
	Medium	0.2076	0.3580	0.4344
	Low	0.7186	0.2346	0.0468
**Light**	High	0.1811	0.2980	0.5209
	Medium	0.3757	0.3357	0.2886
	Low	0.8382	0.1418	0.0200

**Table 8:** Estimation of parameters for modelling on hospital stay.

	*β*_0_	“Stay”	“Res”	“Insured”
		“Long”	“No”	“No”
		*β*_1_	*β*_2_	*β*_3_
Logit(C|A)	−2.885 *** [0.194] (−14.910)	1.230 *** [0.229] (5.366)	3.651 *** [0.236] (15.447)	1.728 *** [0.241] (7.157)
Logit(B|A)	−1.428 *** [0.118] (−12.099)	0.756 *** [0.209] (3.615)	1.682 *** [0.206] (8.170)	1.600 *** [0.219] (7.298)

Residual deviance: 38.46 on 8 d.f.; Log-likelihood: −51.81 on 8 d.f. [s.e.] in square bracket and (z-value) in parentheses. Baseline category for “Stay” = “Short”, “Res” = “Yes”, “Insured” = “Yes”; *** *p* < 0.001.

**Table 9:** Probabilities distribution on time of treatment and status of residency and insurance.

Resident	Insured	Minimally Affected	Adversely Affected	Destitute	Minimally Affected	Adversely Affected	Destitute
		Duration of Hospitalization					
		Long			Short		
**No**	**No**	0.0179	0.2429	0.7392	0.0513	0.3275	0.6212
	**Yes**	0.0901	0.2473	0.6626	0.2253	0.2902	0.4845
**Yes**	**No**	0.2171	0.5495	0.2334	0.3997	0.4747	0.1256
	**Yes**	0.5876	0.3001	0.1122	0.7719	0.185	0.0431

**Table 10:** Estimation results.

	*β*_0_	“Stay”	“Res”	“Insured”	“InsL”			“AvgCost”		“Illness”	
		“Short”	“Yes”	“Yes”	“Lo”	“Med”	“Nil”	“Lo”	“Med”	“Emerg”	“Light”
		*β*_1_	*β*_2_	*β*_3_	*β*_4_	*β*_5_	*β*_6_	*β*_7_	*β*_8_	*β*_9_	*β*_10_
Logit (A|C)	3.122 *** (4.227)	−1.227 *** (−4.569)	−2.644 *** (−9.730)	0.123 (0.209)	1.536 *** (3.432)	0.254 (0.691)	2.224 *** (3.449)	−3.365 *** (−7.866)	−1.584 *** (-4.256)	0.254 (0.919)	−0.992 ** (−3.206)
Logit (B|C)	2.065 ** (2.945)	−0.622 ** (−2.685)	−1.113 *** (−4.686)	−0.282 (−0.520)	0.982 * (2.354)	0.138 (0.434)	1.524 * (2.564)	−1.466 *** (−3.889)	−0.732 c (−1.957)	−0.230 (−0.960)	−1.003 *** (−4.090)

Residual deviance: 1501.93 on 2062 d.f.; Log-likelihood: −750.97 on 2062 d.f. (z-value) in parentheses. Baseline category for “Stay” = “Long”, “Res” = “No”, “Insured” = “No”, “InsL” = “Hi”, “AvgCost” = “Hi”, “Illness” = “Bad”; *** *p* < 0.001, ** *p* < 0.01, * *p* < 0.05, c: *p* < 0.1.

**Table 11:** Modelling extra thank-you money against illness and income rank.

	*β*_0_	“Illness”		“IncRank”
		“Bad”	“Emerg”	“Low”
		*β*_1_	*β*_2_	*β*_3_
Logit (Hi|Neg)	−0.254 [0.202] (−1.270)	−2.106 *** [0.276] (−7.634)	−1.102 *** [0.2699] (−4.082)	−0.896 *** [0.234] (−3.837)
Logit (Med|Neg)	−0.555 ** [0.203] (−2.740)	−0.582 ** [0.220] (−2.641)	−0.316 [0.251] [−1.257]	−0.903 *** [0.195] (−4.628)

Residual deviance: 13.29 on 4 d.f.; Log-likelihood: −33.49 on 4 d.f. [s.e.] in square bracket and (z-value) in parentheses. Baseline category: baseline category for: “Illness” = “Light”, “IncRank” = “HM”; Signif. codes: *** *p* < 0.001, ** *p* < 0.01.

**Table 12:** Probabilities of envelope given by patients’ illness and income rank.

Illness	Negligible	Medium	High	Negligible	Medium	High
	H/M Income			Low Income		
**Light**	0.4256	0.2443	0.3301	0.6455	0.1502	0.2043
**Bad**	0.7066	0.2266	0.0667	0.8558	0.1112	0.0330
**Emergency**	0.5966	0.2496	0.1537	0.7845	0.1330	0.0825

**Table 13:** Estimation of treatment outcome against status of insurance and daily cost.

	*β*_0_	“Insured”	“AvgCost”	
		“No”	“Hi”	“Med”
		*β*_1_	*β*_2_	*β*_3_
Logit(D|A)	−4.106 *** [0. 370] (−11.098)	1.513 *** [0.305] (4.956)	3.526 *** [0.487] (7.239)	1.849 *** [0.420] (4.402)
Logit(C|A)	−5.079 *** [0.589] (−8.617)	1.488 *** [0.344] (4.327)	4.5636 *** [0.666] (6.855)	2.462 *** [0.633] (3.887)
Logit(B|A)	−1.716 *** [0.132] (−13.035)	0.772 *** [0.176] (4.400)	3.119 *** [0.278] (11.208)	1.757 *** [0.173] (10.134)

Residual deviance: 22.49 on 6 degrees of freedom (d.f.); Log-likelihood: −47.83 on 6 d.f. [s.e.] in square bracket and z-value) in parentheses; baseline category for: “Insured” = “Yes”, “AvgCost” = “Lo”; Significance code: *** *p* < 0.001.

**Table 14:** Treatment outcome probabilities conditioned on insurance and average cost.

Insurance	Average Cost	Treatment Outcome			
		Complete Recovery	Self-Care at Home	Stopped	Early Quit
**No**	High	0.0667	0.5873	0.1764	0.1696
	Medium	0.2466	0.5565	0.0797	0.1172
	Low	0.6704	0.2609	0.0185	0.0501
**Yes**	High	0.1606	0.6535	0.0959	0.0900
	Medium	0.4505	0.4695	0.0329	0.0471
	Low	0.8316	0.1495	0.0052	0.0137

**Table 15:** Estimation results of “End” against “Income”.

	*β*_0_	“IncRank”
		“Low”
		*β*_1_
Logit(A|D)	1.509 *** (6.478)	0.018 ** (2.999)
Logit(B|D)	1.748 *** (7.478)	0.003 (0.527)
Logit(C|D)	−0.971 ** (−3.219)	0.018 ** (2.852)

Residual deviance: 2075.56 on 3120 d.f.; Log-likelihood: −1037.78 on 3120 d.f. (z-value) in parentheses; Signif. codes: *** *p* < 0.001, ** *p* < 0.01.

**Table 16:** Distribution of probabilities of patients’ treatment outcomes against their income.

Treatment Outcome	Income (Million VND/year)						
	0	50	100	150	200	400	600
Complete recovery	0.3884	0.5564	0.6993	0.7956	0.8502	0.8932	0.8806
Self-care at home	0.4931	0.3438	0.2104	0.1165	0.0606	0.0036	0.0002
Stopped	0.0325	0.0485	0.0635	0.0752	0.0836	0.1030	0.1192
Early quit	0.0859	0.0513	0.0269	0.0127	0.0057	0.0002	0.0000
